# Validity, Reliability, and Application of the Session-RPE Method for Quantifying Training Loads during High Intensity Functional Training

**DOI:** 10.3390/sports6030084

**Published:** 2018-08-21

**Authors:** Derek A. Crawford, Nicholas B. Drake, Michael J. Carper, Justin DeBlauw, Katie M. Heinrich

**Affiliations:** 1 Department of Health, Human Performance and Recreation, Pittsburg State University, Pittsburg, KS 66763, USA; nbdrake@gus.pittstate.edu (N.B.D.); mjcarper@pittstate.edu (M.J.C.); 2 Functional Intensity Training Laboratory, Department of Kinesiology, Kansas State University, Manhattan, KS 66506, USA; jdeblauw@ksu.edu (J.D.); kmhphd@ksu.edu (K.M.H.)

**Keywords:** perceived exertion, training load monitoring, psychometrics, high intensity functional training

## Abstract

The session rate of perceived exertion method (sRPE) has often been utilized in sports activities in which quantification of external training loads is challenging. The multi-modal, constantly varied nature of high intensity functional training (HIFT) represents a significant hurdle to calculate external work and the sRPE method may provide an elegant solution to this problem. However, no studies have investigated the psychometric properties of sRPE within HIFT interventions. Twenty-five healthy men and women participated in six weeks of HIFT. Rate of perceived exertion and heart rate were assessed within every training session throughout the duration of the intervention. Compared to criterion heart rate-based measures, we observed sRPE method is a valid tool across individual, group, and sex levels. However, poor reliability in participants’ abilities to correctly match rate of perceived exertion with the relative level of physiologic effort (i.e., percentile of maximum heart rate) currently limits the utility of this strategy within HIFT. When applied, the validity and reliability of the sRPE seem to improve over time, and future research should continue to explore the potential of this monitoring strategy within HIFT interventions.

## 1. Introduction

High intensity functional training (HIFT) currently faces criticism on many fronts including medical professionals, academics, and the popular media [1,2,3]. Much of this criticism stems from the proposition that HIFT participation comes with an increased risk of bodily harm [4,5,6,7,8]. However, this contention is largely refuted by evidence showing participation in HIFT is no more dangerous than other common types of exercise activities [9,10,11]. Still, as with other sport and exercise programs, HIFT may benefit from the incorporation of strategies for monitoring and modulating training loads (TL) to optimize outcomes and attenuate any potential of maladaptation [12,13,14].

We have previously shown that participation in HIFT, at least in the short-term, can lead to a functionally overreached state in participants [15]. In sports training, short-term overreaching is often used for brief periods leading up to competition to produce increased performance outcomes [16]. However, to compensate for the time spent with increased TL, periods of overreaching are typically followed by drastically reduced volumes and intensity of training to limit the potential for maladaptation or injury [17]. Central to the ability to modulate training stimuli is the ability to assess TL accurately, and there are a number of different methods to do so including heart rate-based, external load-based, and psychologically-based measures [18].

Due to the constant variance and mixed modality nature of HIFT, monitoring strategies based on external loads are difficult to calculate and meaningfully interpret, thus, limiting their utility in practical settings. Rate of perceived exertion is a well-established metric of an individual’s perceived level of effort during a physical task commonly used to easily quantify TL during sports activities [19,20,21]. Further, using the session rating of perceived exertion method (sRPE) for quantifying TL demonstrates good validity and reliability across various populations and age groups [22]. However, despite its frequent use in other sports, the psychometric properties of sRPE method within HIFT are yet to be determined.

As such, elucidating the validity and reliability of the sRPE method within HIFT might provide athletes and general fitness enthusiasts alike an elegant solution for monitoring and modulating TL within this multimodal exercise program. Thus, the purpose of the present study was to determine the validity and reliability of sRPE for quantifying TL and within HIFT. To achieve this purpose, we investigated the association between sRPE to objectively heart rate-based criterion methods of TL during each training session and the reliability of participants’ perception of workloads. Further, as we have previously shown there is potentially a “learning curve” associated with perceived exertion within HIFT [23], we investigated changes in the validity and reliability of sRPE overtime within a HIFT intervention.

## 2. Materials and Methods

### 2.1. Participants

Twenty-five healthy men (n = 13; mean (Mn.) age = 22.6 ± 3.5; Mn. body mass = 86.1 ± 13.9 kg; Mn. height = 182.8 ± 8.1 cm) and women (n = 12; Mn. age = 21.0 ± 1.5; Mn. body mass = 70.5 ± 11.3 kg; Mn. height = 165.6 ± 5.7 cm) participated in the present study. Participants were recreationally active but without prior experience in HIFT programs. All participants reported no presence of any health conditions prohibiting participation in high-intensity exercise. Participants’ provided their informed written consent following familiarization with study protocols which had been approved by a university Institutional Review Board in accordance with the Declaration of Helsinki.

### 2.2. Experimental Design

This study is a secondary analysis of a previous investigation performed over a nine-week period to assess the validity of the sRPE method for quantifying TL during HIFT [24]. Participants completed 5 d·wk^−1^ of HIFT (i.e., Monday–Friday) at a local facility conducive to HIFT practices. Rate of perceived exertion and heart rate were measured within each training session. For all current study analyses, data from weeks two, three, four, six, seven, and eight of the previous investigation are used (i.e., 6 weeks of HIFT in total). All training sessions were guided and supervised by a trained HIFT practitioner with a bachelor’s degree in kinesiology. Pre-training data (i.e., participant demographics and anthropometrics) were obtained in a laboratory setting after participants consented to study protocols.

### 2.3. High-Intensity Functional Training Intervention

The specific intervention utilized in the present study followed a popular, community-based template of HIFT [25]. The exact details for the “Daily Workout” (DW) for each training session can be seen in Table A1 within the previously published work [24]. Each session was approximately 1 h in duration and included a brief warm-up, the programmed DW, and some mobility work as a cool-down. Participants were instructed to give maximal effort/intensity for each DW throughout the intervention protocol. Participants were scheduled to complete 30 HIFT sessions during the six-week period, and participant adherence to the intervention was 87.9 ± 8.3%. Participants were allowed to remain in free-living conditions throughout the study duration but were asked to avoid any supplemental exercise activity outside of their prescribed HIFT sessions.

### 2.4. Rate of Perceived Exertion

Participants reported their rate of perceived exertion approximately five minutes following the completion of each DW throughout the study duration. Rate of perceived exertion was assessed using the original Borg’s Rate of Perceived Exertion scale (i.e., 6–20; *very very light* to *very very hard*) [26,27] and participants were asked to report their “overall” exertion during the DW.

### 2.5. Heart Rate

Training heart rate (HR) was assessed using a commercially-available continuous heart rate monitor (Polar H7^®^ heart rate monitor, Polar Electro, Inc., Bethpage, NY, USA) worn across the chest during each training session. Data were collected continuously during each DW using the manufacturer-developed smartphone application (Polar Beat^®^ version 2.3.2., Polar Electro, Inc., Bethpage, NY, USA). Immediately following the completion of the daily DW, participants stopped data collection and reported both the duration (in minutes) and average HR observed during their session from the smartphone application. This data was reported following the collection of participants’ rate of perceived exertion.

### 2.6. Session-RPE Method for Quantifying Training Load

Daily TL for each training session was calculated using the session RPE method (sRPE) [14]. This method involved calculating the product of the DW duration in minutes by the perceived training intensity measured by Borg’s rating of perceived exertion scale noted above.

### 2.7. Criterion Methods of Quantifying Training Loads

Two established methods of HR-based quantification of TL were used as criterion measures within this investigation. These criterion methods include the Edwards’ internal workload [28] (Edwards’ TL) and Banister’s Training Impulse [29] (TRIMP) quantifications. The Edwards’ TL was determined by integrating the total volume (i.e., duration in minutes) of exercise with the total intensity of each HIFT session relative to five pre-determined intensity thresholds. Each pre-determined heart rate zone had its own unique factor (50–60% HRmax = 1; 60–70% HRmax = 2; 70–80% HRmax = 3; 80–90% HRmax = 4; 90–100% HRmax = 5) which was multiplied by the total duration spent within each of these zone and then summing the results [19]. TRIMP weights the duration of each training session duration using the following formula:

Training Load = D × HRr × 0.64e^1.92 × HRr^

where D = duration in minutes and HRr is determined by HRts − HRb)/(HRmax − HRb), where HRb is the individual’s resting heart rate and HRts is the average heart rate observed over the training session [30].

### 2.8. Statistical Analyses

All data were tested for normality before completion of inferential statistical tests. To investigate criterion validity, Pearson’s *r* correlation coefficients were used to determine the association between the sRPE-method and the HR-based criterion measures at the group, individual, and gender levels. The within-subject rate of perceived exertion and average training HR quartiles (i.e., 0–25%, 26–50%, 51–75%, and 76–100%) were calculated to investigate intra-rater reliability. The agreement in participants’ reporting of these two variables (i.e., correct matching of rate of perceived exertion and average HR quartiles within a given training session) was assessed using the interclass correlation coefficient and coefficient of agreement (CoA). Linear regression between sRPE and HR-based criterion measures were used to investigate potential changes in the association between these variables over time (i.e., weeks 1–3 versus weeks 4–6 of training). All regression analyses presented were checked for violation of multicollinearity (i.e., Watson-Durbin statistic between 1.5–2.5) and homoscedasticity assumptions. All inference testing was performed using SPSS v. 24 (IBM Inc., Armonk, NY, USA). An alpha level of 0.05 was used for all null hypothesis testing. Supporting statistical information including *p*-values, effect sizes, and 95% confidence intervals (95% CI) were reported were appropriate. 

## 3. Results

### 3.1. Validity of Session-RPE Method to HR-Based Criterions

Table 1 illustrates the Pearson *r* correlation coefficients between sRPE method and Edwards’ TL and TRIMP quantifications. Coefficients are presented at the individual, group (i.e., overall), and sex levels. Overall, at the group level, sRPE was significantly correlated with both the Edwards’ TL (n = 558, *r* = 0.889; *p* < 0.0001) and TRIMP (n = 554, *r* = 0.561; *p* < 0.0001) calculations. At the individual-level, sRPE and Edwards’ TL was significantly correlated (*r* = 0.677–0.973; all *p* < 0.001) with 100% of individuals demonstrating a significant association. In contrast, the association between sRPE and TRIMP, while significant (*r* = 0.279–0.831; 0.05 < *p* < 0.001), was only present for 92% of participants. Significant associations remained present between sRPE and criterion methods for both sexes.

### 3.2. Reliability of Session RPE for Predicting Average Heart Rate

Intra-rater reliability was determined using the agreement between participants’ reported perception of effort (i.e., rate of perceived exertion quartile) and physiologic effort (i.e., average HR quartile) within training sessions. Quartiles were created at the individual-level to control for potential variations in the sRPE-to-HR relationship due to baseline fitness status (i.e., participants reporting differing levels of effort for a similar percentage of maximum heart rate) [31]. While the interclass correlation between these variables was statistically significant (n = 554, ICC = 0.586, 95% CI = 0.529–0.638, *p* < 0.001), interclass correlation coefficient (ICC) values below 0.75 generally indicate *poor* to *moderate* reliability [32]. Further, the CoA (i.e., proportion of correct agreements/the total number of possible agreements) of only 52% agreement between rate of perceived exertion and HR quartiles, again, illustrates low reliability.

### 3.3. Application of Session-RPE Method Throughout a HIFT Intervention

**Figure 1:**
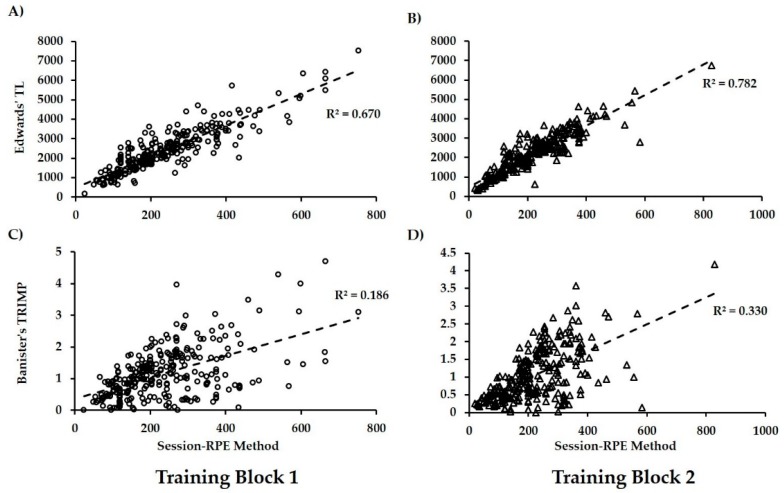
Scatterplots for regression analyses of session rate of perceived exertion (sRPE) for prediction heart rate-based criterion methods. (**A**) sRPE vs. Edwards’ internal workload (Edwards’ TL) for Block 1; (**B**) sRPE vs. Edwards’ TL for Block 2; (**C**) sRPE for Banister’s Training Impulse (Banister’s TRIMP) for Block 1; (**D**) sRPE for Banister’s TRIMP for Block 2. All analyses significant at *p* < 0.001.

The relationship between the sRPE and HR-based criterion methods was investigated for two separate 3-week training blocks (i.e., Block 1 = weeks 2–4, Block 2 = weeks 6–8) within the HIFT intervention. Figure 1 shows linear regression scatterplots for sRPE predicting Edwards’ TL and TRIMP for both Block 1 and Block 2.

For Block 1, sRPE significantly predicted both Edwards’ TL (n = 271, *r* = 0.818, *p* < 0.001, 95% CI for R^2^ = 0.606–0.732) and TRIMP (n = 260, *r* = 0.431, *p* < 0.001, 95% CI for R^2^ = 0.101–0.270). For Block 2, these regressions remained significant but also improved in their predictive capability for both Edwards’ TL (n = 268, *r* = 0.885, *p* < 0.001, 95% CI for R^2^ = 0.733–0.826) and TRIMP (n = 258, *r* = 0.575, *p* < 0.001, 95% CI for R^2^ = 0.230–0.423). Potential changes in intra-rater reliability of participants’ rate of perceived exertion and HR quartiles was also investigated between training blocks. The interclass correlation coefficient for Block 2 (n = 270, ICC = 0.613, 95% CI = 0.533–0.683, *p* < 0.001) was improved compared to Block 1 (n = 284, ICC = 0.560, 95% CI = 0.529–0.638, *p* < 0.001) and, similarly, the CoA also improved (57% vs. 47%, respectively).

## 4. Discussion

This study is the first to investigate the psychometric properties of the sRPE method within HIFT. The sRPE method appears to be a valid tool for quantifying TL within HIFT as evidenced by its strong association with HR-based criterion methods at both the individual and group levels as well as across genders. However, the intra-rater reliability for correctly matching rate of perceived exertion with relative workload (i.e., percentage of maximum heart rate) is generally poor. However, over time, participants are able to improve the reliability of their perceptions of relative workload during HIFT sessions which may be responsible for the improvement in the association of the sRPE method and criterion measures as noted within the present study.

The significant associations between sRPE, Edwards’ TL, and TRIMP lend support to the criterion validity of this monitoring strategy within HIFT. As others have reported, sRPE is particularly useful in sports activities where quantification of external loads is often difficult [19,22,30]. We propose that the multi-modal nature of HIFT requires that participants and practitioners looking to moderate training prescriptions within this exercise program will likely need such a method. Further, in contrast to the above-referenced investigations, these data indicate that this method is valid across individuals and sexes similar to recent work highlighting its validity for different age groups and populations [22]. While the significant associations present within this study are exciting, one must also consider the reliability of individuals’ ability to accurately perceive their level of effort within HIFT must also be of equal importance.

While we report positive findings with respect to the validity of the sRPE method compared to criterion methods, unfortunately, the reliability of this method within HIFT is not as encouraging. These data indicate, within the present sample, participants were unable to accurately perceive their level of effort relative to the percentage of maximum heart rate achieved during training sessions. Both the interclass correlation coefficient (i.e., ICC = 0.583) and coefficient of agreement (i.e., 52%) indicate these participants were poor at matching the relative quartile of rate of perceived exertion with the corresponding relative quartile of percentage heart rate (e.g., correctly reporting 75th percentile of perceived exertion when training within 75th percentile of heart rate). In a pilot investigation, we have previously shown that novice participants may incorrectly perceive their level of effort early within HIFT interventions [22]. While the novelty of HIFT compared to other exercise modalities could contribute to the poor reliability within this study, evidence also suggests that within the higher intensity exercise domains (e.g., heavy or severe) individuals already have difficulty in reliably perceiving their level of physiological effort [33]. Further, even in more controlled exercise tasks (e.g., intermittent running at different intensities), individuals demonstrate poor reliability test–retest reliability in reporting rate of perceived exertion [34]. Despite this poor reliability, Scott et al. (2013) contend that the sRPE method is still a valid tool for monitoring TL in sport. However, if the critical element of a monitoring strategy (i.e., the surrogate measure of effort/load) is not able to be reliability reported, we contend that the monitoring strategy is largely invalid [35] within this current study of HIFT. However, identifying strategies such as attentional focus [36] or implementation planning [37] to assist HIFT participants in improving the reliability of their rate of perceived exertion during HIFT may rectify this issue.

In our pilot investigation, we noted that HIFT participants’ perception of effort became more accurate for predicting TL (i.e., Edwards’ TL) after a period of three weeks [23]. Within the present study, this phenomenon holds true as we observed improvements in both the predictive validity and intra-rater reliability of the sRPE method between Block 1 (i.e., the first three weeks of training) and Block 2 (i.e., second three weeks of training). We contend that the improvement in validity, while not statistically assessed, is potentially meaningful as evidenced by the non-overlapping confidence intervals for the R^2^ value for sRPE and Edwards’ TL between Block 1 and 2. In addition to this improvement in validity, we also observed potentially meaningful improvement in the reliability of rate of perceived exertion for correctly identifying physiologic effort between Block 1 (i.e., ICC = 0.530; CoA = 47%) and Block 2 (i.e., ICC = 0.613; CoA = 57%). Unfortunately, no studies investigating longitudinal changes in rate of perceived exertion within exercise interventions could be identified. As such, it is challenging to elucidate the reasoning for this improvement. However, within the present sample, we advance the proposition that the observed improvement of validity and reliability of the sRPE method may be mediated through reductions in perceived exertion (*t* = 1.94; *p* = 0.053) between Block 1 (Mn. = 15.4 ± 3.7) and Block 2 (Mn. = 14.9 ± 3.3) even though TL does not change significantly (e.g., Mn. difference in Edwards’ TL = 124.2 ± 175.6, *F* = 1.92, *p* = 0.166, η^2^ = 0.003). We noted a similar effect in our pilot investigation wherein rate of perceived exertion decreased even as TL increased over the intervention [23]. Future investigations should look to experimentally test this hypothesis.

Strengths of the current study are the inclusion of multiple criterion measures (i.e., both Edwards’ TL and TRIMP), assessment of reliability characteristics between participants’ rate of perceived exertion and physiologic effort, and examination of validity across the individual, group, and sex levels. However, there are limitations worth noting if future researchers aim to replicate these findings. First, we only assessed rate of perceived exertion at one time point within the exercise sessions (i.e., immediately following completion of the daily DW). It is entirely plausible that the level of exertion perceived at this time point is significantly higher than what may have been reported over the duration of the exercise session due to the “end spurt” phenomena and potential pacing strategies utilized by participants during HIFT [38]. Assessing rate of perceived exertion at multiple time points within exercise sessions may allow for the creation of a more global representation of participant exertion that in of itself may improve the validity and reliability of the sRPE method. Second, while the present study employed criterion measures of internal TL (i.e., HR-based measures) it would be equally important to investigate the validity of sRPE with external quantification of TL within HIFT. While difficult to calculate, and, thus, impractical for real-world settings, researchers can determine the amount of external work (e.g., mechanical power) completed during HIFT sessions. Calculations of mechanical power may be more sensitive in monitoring TL as HIFT often employees training sessions that focus on heavy weightlifting activities (e.g., working up to a 3-repetition maximum deadlift over seven sets) that traditional HR-based measures may identify as “lower” effort. Third, controlling for preferences of exercise intensity [39] or baseline fitness status (e.g., maximal aerobic capacity) may allow for an easier statistical methodology for mitigating any potential confounding effects of these variables on the RPE and HR relationship. Lastly, the methodology for classifying HR zones for reliability and agreement assessments used within the present study is novel. As such, there could be alternative methods (e.g., narrower HR zones; 70–90% vs. 75–100%) which may potentially affect the results presented here. Future research should look to address these limitations along with investigating the strategies that may improve the reliability of participants’ RPE during HIFT.

## 5. Conclusions

HIFT interventions are predicated on participants providing maximal effort on a daily basis. Quantification of external training loads remains challenging, and those interested in moderating or modulating training stimuli may find it feasible to implement a strategy such as perceived effort. This is the first study to investigate the validity, reliability, and application of the sRPE method within HIFT. While the sRPE method is a valid strategy for monitoring TL when compared to HR-based criterion measures, the inability of participants to reliably match their perceived exertion with the level of physiologic effort (i.e., percentile of HR maximum) currently limits the utility of the sRPE method within HIFT. However, as participants become more accustomed to HIFT, we observed that both the validity and reliability of this method improves. Future research should test strategies for improving the reliability of perceived exertion relative to physiologic effort before employing the sRPE method within real-world HIFT practice.

## Figures and Tables

**Table 1:** Pearson *r* correlations for session rating of perceived exertion (sRPE) at the group, individual, and gender levels.

Participant	Sex	Edwards‘ TL	Banister’s TRIMP
01	M	0.920 **	0.619 **
02	M	0.954 **	0.669 **
03	M	0.872 **	0.751 **
04	M	0.871 **	0.722 **
05	M	0.924 **	0.656 **
06	M	0.946**	0.523 **
07	F	0.973 **	0.831 **
08	M	0.907 **	0.803 **
09	M	0.794 **	0.292
10	M	0.949 **	0.638 **
11	M	0.797 **	0.279
12	F	0.858 **	0.629 **
13	F	0.911 **	0.411 *
14	F	0.891 **	0.564 **
15	M	0.922 **	0.466 *
16	F	0.963 **	0.651 **
17	F	0.973 **	0.788 **
18	F	0.878 **	0.554 *
19	F	0.962 **	0.715 **
20	F	0.920 **	0.690 **
21	M	0.820 **	0.441 *
22	F	0.955 **	0.782 **
23	M	0.858 **	0.483 *
24	F	0.677 **	0.536 *
25	F	0.888 **	0.456 *
**Overall**	-	0.889 **	0.561 **
*Females*	-	0.896 **	0.611 **
*Males*	-	0.883 **	0.559 **

* Significant correlation at *p* < 0.05, ** Significant correlation at *p* < 0.001, M = male, F = female.
